# Decline of *FOXN1* gene expression in human thymus correlates with age: possible epigenetic regulation

**DOI:** 10.1186/s12979-015-0045-9

**Published:** 2015-10-29

**Authors:** Maria Danielma dos Santos Reis, Krisztian Csomos, Luciene Paschoal Braga Dias, Zsolt Prodan, Tamas Szerafin, Wilson Savino, Laszlo Takacs

**Affiliations:** Laboratory on Thymus Research, Oswaldo Cruz Institute, Oswaldo Cruz Foundation, Rio de Janeiro, Brazil; Department of Human Genetics, Faculty of Medicine, University of Debrecen, Nagyerdei krt. 98, H-4032 Debrecen, Hungary; Department of Biochemistry and Molecular Biology, Faculty of Medicine, University of Debrecen, Debrecen, Hungary; Gottsegen György Hungarian Institute of Cardiology, Budapest, Hungary; Department of Cardiac Surgery, Clinical Center, University of Debrecen, Debrecen, Hungary; Biosystems International Kft, Debrecen, Hungary

## Abstract

**Background:**

Thymic involution is thought to be an important factor of age related immunodeficiency. Understanding the molecular mechanisms of human thymic senescence may lead to the discovery of novel therapeutic approaches aimed at the reestablishment of central and peripheral T cell repertoire.

**Results:**

As an initial approach, here we report that the decline of human thymic *FOXN1* transcription correlates with age, while other genes, *DLL1, DLL4* and *WNT4*, essential for thymopoiesis, are constitutively transcribed. Using a human thymic epithelial cell line (hTEC), we show that FOXN1 expression is refractory to signals that induce *FOXN1* transcription in primary 3D culture conditions and by stimulation of the canonical WNT signaling pathway. Blockage of FOXN1 induceability in the hTEC line may be mediated by an epigenetic mechanism, the CpG methylation of the *FOXN1* gene.

**Conclusion:**

We showed a suppression of *FOXN1* transcription both in cultured human thymic epithelial cells and in the aging thymus. We hypothesize that the underlying mechanism may be associated with changes of the DNA methylation state of the *FOXN1* gene.

**Electronic supplementary material:**

The online version of this article (doi:10.1186/s12979-015-0045-9) contains supplementary material, which is available to authorized users.

## Background

In human, the thymus-derived naïve T cell repertoire, capable to exert effective protection to foreign antigens, is established during early embryonic life and it reaches maximal size in childhood [1, 2], subsequently, as antigen specific T cells are generated, the naïve T cell pool is gradually depleted. Thus, the limited naïve T-cell repertoire in elderly individuals is a major contributor to age-related immunodeficiency, a frequent cause of death [3, 4]. The immune compromised status results in the lack of effective immune response against pathogenic microrganisms and malignant cells. Because age related immunodeficiency is often life limiting as the cause of frequent nosocomial infections of the elderly, and because current treatment is insufficient, moreover it represents a significant medico-economic burden [5], there is a strong interest to develop effective and economically sound therapies. One possible strategy is the restoration of the naïve T cell repertoire via therapeutic regeneration of thymic activity.

Bone marrow derived stem cells migrate to the thymus where they proliferate and differentiate to T cell receptor (TCR) expressing T cells while their progeny centripetally migrate in, and eventually exit the organ. Accordingly, the recent emigrant naïve T cells permanently contribute to the peripheral T cell to maintain TCR repertoire diversity, and, at least in part, age related immunodeficiency is the result of the decline of naïve emigrant T cell production [6]. Intrathymic T cell development is orchestrated by the microenvironment, a meshwork composed of stromal cells, such as dendritic cells, fibroblasts, macrophages and thymic epithelial cells (TEC), as well as by the extracellular matrix (ECM) molecules, which provide a unique three-dimensional environment [7]. The thymic stromal cells are distributed within the thymic epithelial space, which is divided into two main compartments, cortex and medulla [8, 9]. In the cortical and medullary microenvironments, TEC interact with developing thymocytes via cell surface receptors, the production of ECM molecules, cytokines, chemokines and growth factors [10]. Thymic epithelial cells express (i) notch ligands which direct and restrict the bone marrow precursors to the T cell differentiation program [11] and (ii) self-antigen filled major histocompatibilty complex molecules (MHC) which serve as substrates for TCR repertoire selection [12].

In humans, as the thymus ages, thymic epithelial mesh is gradually replaced by adipose tissue. The process is thought to start at the first year of life and continues during aging [8, 13], being accompanied by a decreasing export of naive T cells [14]. The underlying molecular mechanisms responsible for the impairment of thymopoiesis in the aging thymus remains unclear. One possibility is that intrinsic mechanisms related to TEC physiology are impaired in old individuals, since bone marrow precursors from old animals are able to colonize the thymus [15]. In fact, some studies showed that TEC proliferation is lower in old animals and it was also demonstrated that aging mice have higher percentage of apoptotic and senescent TECs [15, 16].

Studies in rodent models pointed out that the transcriptional factor forkhead box protein N1 (FOXN1) is both necessary and seemingly sufficient to induce differentiation of functional TEC [17, 18]. FOXN1 appears on day 11 during mouse embryonic development, the sixth week of gestation in humans, and induces the thymic organogenesis program presumably under the control of WNT family of glycoproteins, namely, by WNT-4 [2, 19, 20]. In a model with inducible Cre mediated deletion of an SV40 driven transgenic hypomorphic *Foxn1* allele, it has been demonstrated, that FOXN1 in TEC induces the expression of MHC II, CD40, PAX1, cathepsin-L, the chemokine CCL25 and the NOTCH ligand Delta-like 4 (DLL4), thus highlighting its orchestrating role in T cell maturation [21]. The lack of FOXN1 in mice and rats results in the absence or the incomplete development of TEC and the thymic epithelial mesh, combined with severe immunodeficiency known as the nude phenotype [22]. Nude mice carry a single base pair deletion at exon 3 of the *Foxn1* gene, which results in aberrant protein production, lacking the DNA-binding and the transcription activation domains, necessary for FOXN1 protein function [23, 24]. Similar phenotype was found in human, carrying a rare non-sense mutation at the residue 255 of the FOXN1 protein, resulting from a single base substitution in exon 5 of the *FOXN1* gene [25]. Recently, Bredenkamp and co-workers showed that mouse embryonic fibroblasts transfected with inducible *Foxn1* transdifferentiated to functional TECs upon induction [18]. These *Foxn1* induced TECs support T cell development *in vitro* and *in vivo*. The data clearly demonstrate the central role of FOXN1 in thymic epithelial function and organogenesis. Murine models showed a decrease on *Foxn1* expression in aged thymus [26, 27]. Others found that the decline of *Foxn1* expression results in a decrease of thymus cellularity and function, compared to normal aged murine thymus [28, 29]. In contrast, thymus from aged mice, with high transgenic *Foxn1* levels, presented morphology and T cell maturation similar to the thymus of young mice [30]. In addition, it was demonstrated that induction on *Foxn1* expression in a murine aging model was able to restore thymic architecture and T cell export, similar to pre-involuted thymus [31]. Despite the overwhelming genetic [32] and molecular evidence pointing to the *FOXN1* gene as the key regulator of thymopoiesis is aging, little is known on human FOXN1 function and its regulation.

As an initial approach, from consented, young and aging donors thymic biopsy specimens and a human postnatal TEC line (hTEC) [33], we tested the expression of genes that have been reported to be essential for T cell development, particularly the inducibility of the *FOXN1* gene expression. Subsequently, we tested the methylation status of predicted transcriptional regulatory regions of the human *FOXN1* gene inthe cultured human TEC line.

## Results

### Expression of *DLL1, DLL4, FOXN1* and *WNT-4* genes in human thymic samples

**Fig. 1:**
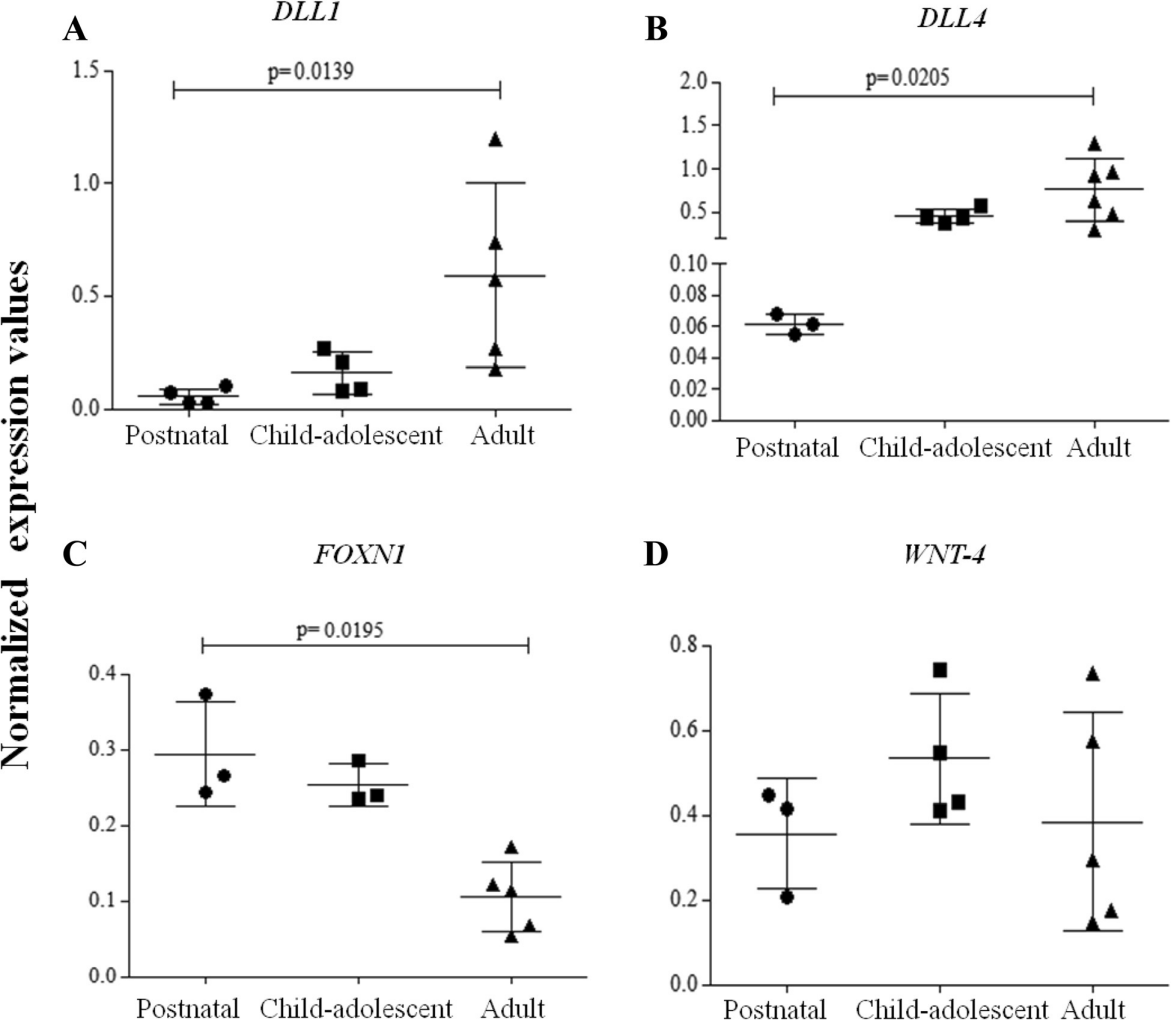
*DLL1, DLL4, FOXN1* and *WNT-4* gene expression in human thymic samples. Gene expression was assessed by real-time quantitative PCR. *DLL1* (**a**), *DLL4* (**b**), *FOXN1* (**c**) and *WNT-4* (**c**). The graphs show mean ± SEM of normalized expression values from each group. Difference between the samples was analyzed through Kruskal-Wallis non-parametric test followed by Dunn’s multiple test. Data was considered statistical significant when *p* < 0.05

Thymic samples were divided into three different groups according the donor’s age. The groups were created by considering major hormonal transitions as shown previously [34]. The “Postnatal” group comprises samples from 5 days-old to 1 year-old (a total of four samples); the “Child-adolescent” group contains samples whose donors are 7, 10, 14 and 17 years-old (a total of four samples); the “Adult” group included samples from donors, 49, 57, 59, 66, 75 and 78 years-old (seven samples total). As represented in Fig. 1a and b, respectively, the Delta-like 1 (*DLL1*) and 4 (*DLL4*) genes presented low relative levels in the younger groups, showing a significant elevation in the “Adult” group, while the Forkhead box protein N1 (*FOXN1*) gene expression was significantly decreasing from “Postnatal” through “Child-adolescent” to the “Adult” group (Fig. 1c). It is important to note that age vs. normalized expression levels of *FOXN1* do correlate significantly (see Additional file 1: Figure S1). The wingless-type MMTV integration site family, member 4 (*WNT-4*) gene expression seemed constitutive, not showing remarkable differences over time (Fig. 1d).

### Histology, thymic epithelial organization and FOXN1 protein expression

**Fig. 2:**
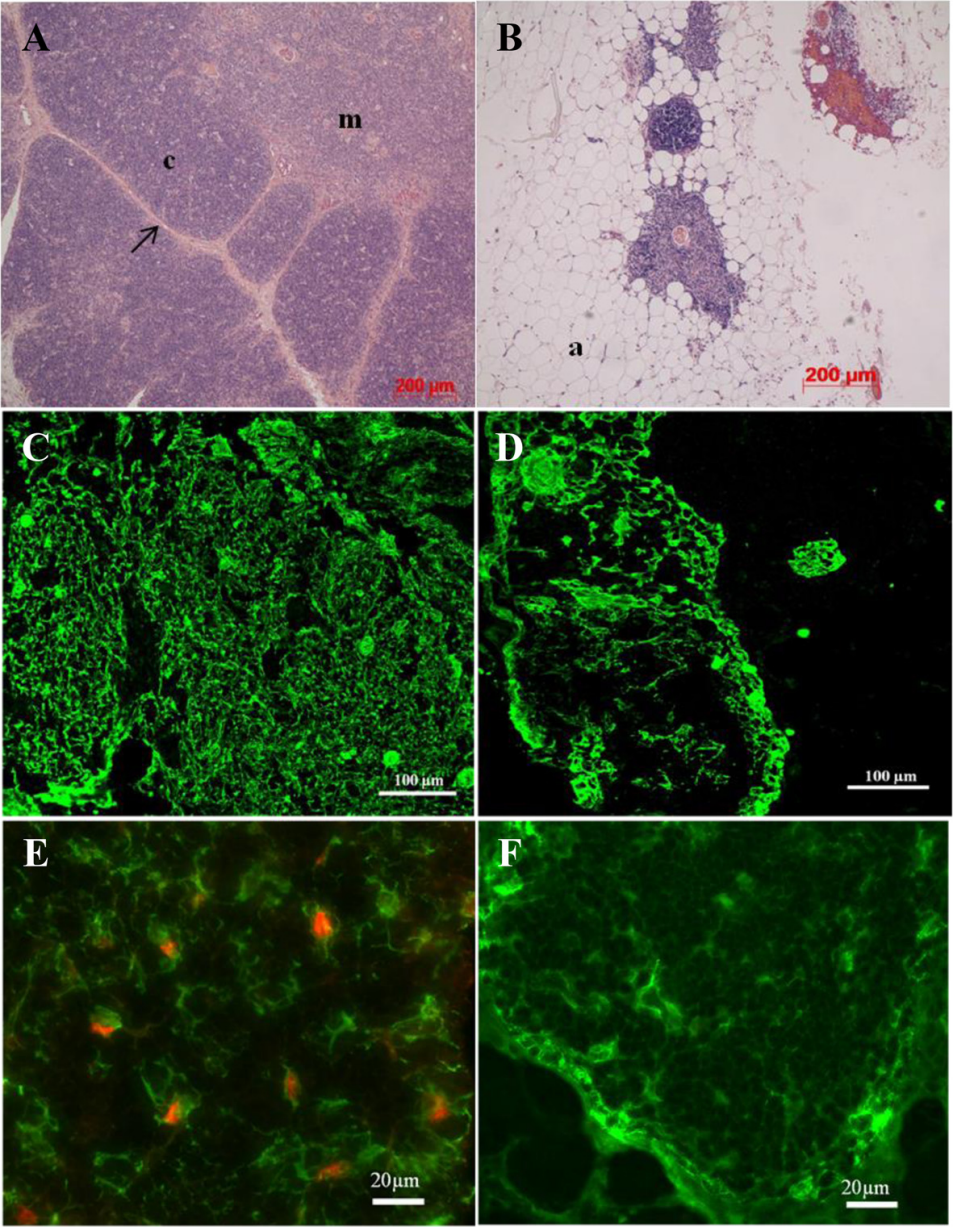
Histology, thymic epithelial organization and FOXN1 protein expression. Photomicrographs show morphological aspects of thymus samples from (**a**) five days (“Postnatal” group) and (**b**) 57 years-old donors (“Adult” group). Paraffin sections were stained with haematoxilin-eosin stain and analyzed by light microscopic examination. (4x, objective magnification). c = cortical region; m = medullary region; a = Adipose tissue; arrow = connective tissue septum. Panels (**c**) and (**d**) show cytokeratin staining of “Postnatal” (5 days-old) and “Adult” thymic samples (57 years-old), respectively. Frozen thymus sections were reacted with specific pan anti-cytokeratin polyclonal rabbit antibody followed by Alexa-488 conjugated anti-rabbit Ig antibody (green) before visualization in the fluorescent microscope. (20x, objective magnification). Photomicrographs (**e**) and (**f**) show FOXN1 protein expression in thymus cryosections from “Postnatal” (5 days-old) and “Adult” donors (57 years-old), respectively. In order to demonstrate thymic epithelium associated FOXN1 expression, the sections were first reacted with anti-human FOXN1 antibody (red) and subsequently double stained with pan anti-cytokeratin antibody (green). (20x, objective magnification)

The “Postnatal” thymic samples, from 5 days-old to 1 year-old, showed typical morphology, with the surrounding connective capsule, trabeculae, cortical and medullary regions. With the exception of occasional individual fat cells in the interlobular areas, we did not detect thymic adipose tissue in these samples (Fig. 2a). Cytokeratin labelling pattern revealed the typical thin mesh distributed throughout the thymic cortex, and with more dense medullary TEC (Fig. 2c). In the “Child-adolescent” group we detected considerable amount of organized adipose tissue embedded in interlobular areas (See Additional file 1: Figure S2). It is important to note that the cortex to medulla ratio is lower, thus the thymic lobes seem to possess relatively larger medullary areas and overall, smaller lobes (See Additional file 1: Figure S2). In the “Adult” group, intrathymic adipose tissue dominates, and no well-defined cortical and medullary regions could be distinguished (Fig. 2b). The cytokeratin organization is severely altered, and this becomes more profound with increasing age (Fig. 2d).

Since with qPCR we found significantly lower *FOXN1* gene expression in the “Adult” group compared to the younger groups of thymic samples, we tested and compared the expression of FOXN1 protein in samples from the “Postnatal” and “Adult” groups by immunofluorescence. We observed characteristic nuclear FOXN1 staining in the “Postnatal” group. FOXN1 positive cells were distributed in the cortex, although the majority of epithelial cells were negative, suggesting heterogeneity of thymic epithelium with respect to FOXN1 expression (Fig. 2e). Surprisingly, no FOXN1 protein expression was detectable in samples from the “Adult” group, even though the thymic epithelium was clearly detectable, typical staining patterns are shown on Fig. 2f.

### hTEC cell line, a model of aging thymic epithelium

**Fig. 3:**
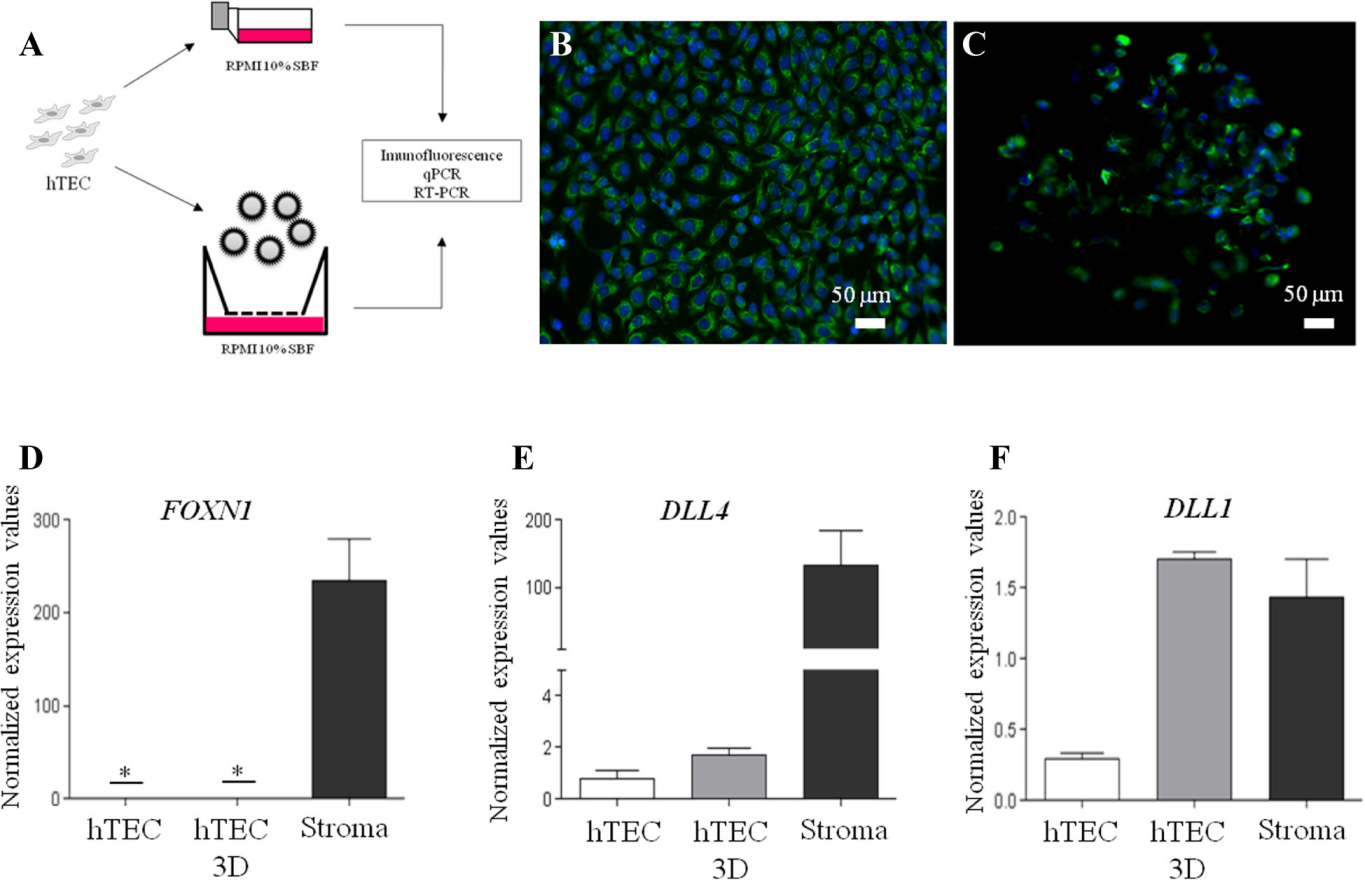
hTEC line, a model of aging thymic epithelium: three-dimensional cell culture in cellulose scaffolds. Conventional hTEC monolayer culture (**b**) and hTEC 3D culture (**c**) maintained for four days in cellulose macroporous scaffolds were immunocytochemical staining for cytokeratin with DAPI nuclear counterstaining. (20x, objective magnification). qPCR detection of *FOXN1* (**d**), *DLL4* (**e**) and *DLL1* (**f**) transcripts, in conventional monolayer and 3D cultures. Thymic stroma from 5 days-old sample served as control (“Stroma”). Undetectable level of expression is labelled with “*”

Recent reports indicate that thymocyte development is induced and supported by *Foxn1* transduced mouse fibroblast cells that transdifferentiate to functional thymic epithelium [18]. Thymus derived epithelial cell lines and primary monolayers of TEC cultures do not induce full scale T cell development [35, 36], and this may be related to a decrease of FOXN1 expression [19, 37] by a yet unidentified mechanisms. A human TEC line (hTEC), which expresses membrane proteins specific for human TEC (*in vivo*), and able to adhere to T cell precursors, immature and mature naïve T cells was chosen here for further investigations [33]. In preliminary experiments, we found no *FOXN1* mRNA expression in hTEC cells (Fig. 3d). Nevertheless, other tested thymic epithelium genes that are necessary for T cell development were expressed (Fig. 3e and f). Due to the lacking *FOXN1* expression, the striking gene expression pattern similarity with the aging human thymus, the hTEC line served as a model of the aging thymic epithelium here. Using this model we tested conditions that have been reported to induce FOXN1 expression [19, 37]. First, we prepared three-dimensional (3D) cultures of hTEC (Fig. 3a) and found that both conventional monolayer and 3D hTEC cultures expressed and maintained epithelial characteristics as ascertained by cytokeratin expression (Fig. 3a and b, respectively). However, neither conventional nor 3D cultures expressed detectable *FOXN1* transcripts, despite the fact that the 3D culture system based on microspherical substrate did function, since *DLL4* and *DLL1* transcripts were detectable and showed remarkable induction by the 3D culture conditions (Fig. 3d, e and f). It is worth to note that we tested T cell development inducing/maintaining capacity of the hTEC by co-culturing thymocytes, both in monolayer and in 3D cultures, but we did not observed increased thymocyte production in the cultures (data not shown).

**Fig. 4:**
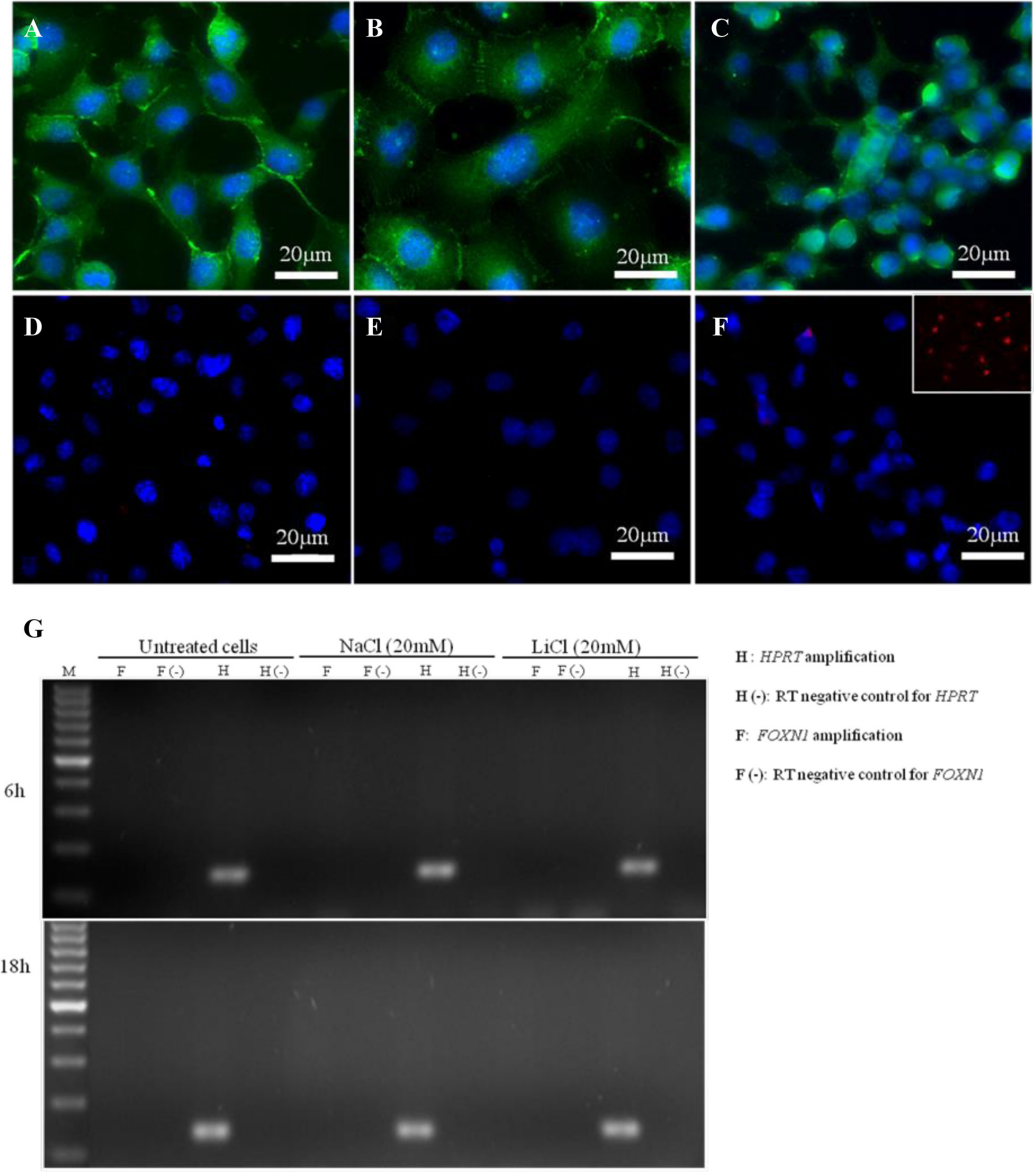
hTEC line, a model of aging thymic epithelium. WNT signaling pathway induction. Conventional hTEC monolayer cultures stained with anti-β-catenin monoclonal antibody (green) (**a**–**c**) and anti-human FOXN1 (red) (**d**–**f**) with DAPI nuclear counterstain: non-treated control (**a**, **d**), LiCl-treated for 6 h (**b**, **e**) and 18 h (**c**, **f**). (40x objective magnification). FOXN1 staining on a thymic human sample was used as a positive control (Fig. 4f, insert). In (**g**) is shown the RT-PCR detection of *FOXN1* transcript after treatment with 20 mM of LiCl for 6 and 18 h. Non-treated cultures and TEC treated with 20 mM of NaCl were used as negative controls. Legend – F: *FOXN1* amplification product; F (−): reverse transcriptase reaction control without enzyme from *FOXN1* amplification product; H: *HPRT-1* amplification product; H (−): reverse transcriptase reaction control without enzyme from *HPRT-1* amplification product

In thymus-derived primary epithelial cultures, lithium chloride (LiCl), a glycogen synthase kinase-3β inhibitor and a mimetic of the canonical WNT-4 signaling pathway [38], was shown to induce *Foxn1* transcription, which was accompanied by β-catenin translocation to the nucleus [19]. Here, we tested whether LiCl induced FOXN1 expression in hTEC line (Fig. 4). Upon exposure of hTEC cultures to LiCl treatment for 6 h and for 18 h, characteristic morphological changes were observed, namely intensive cell spreading at 6 h (Fig. 4b) and overgrowth, apoptotic aggregation of cell clusters at 18 h (Fig. 4c), with accompanied β-catenin reorganization, which included detectable β-catenin nuclear translocation at in the 6 h of culture (Additional file 1: Figure S3). As LiCl induced the expected biological response in hTEC, we tested FOXN1expression using immunostaining and RT-PCR. FOXN1 protein expression was not detectable on untreated hTEC (Fig. 4d), in agreement with the qPCR data showed on Fig. 3c. Upon LiCl treatment, the *FOXN1* gene remained silent, with no detectable FOXN1 protein in the LiCl-treated hTEC cultures (Fig. 4e and f) and no detectable transcripts produced in the control or in LiCl-treated cell cultures (Fig. 4g).

### FOXN1 genomic context of candidate CpG methylation substrate, C20

**Fig. 5:**
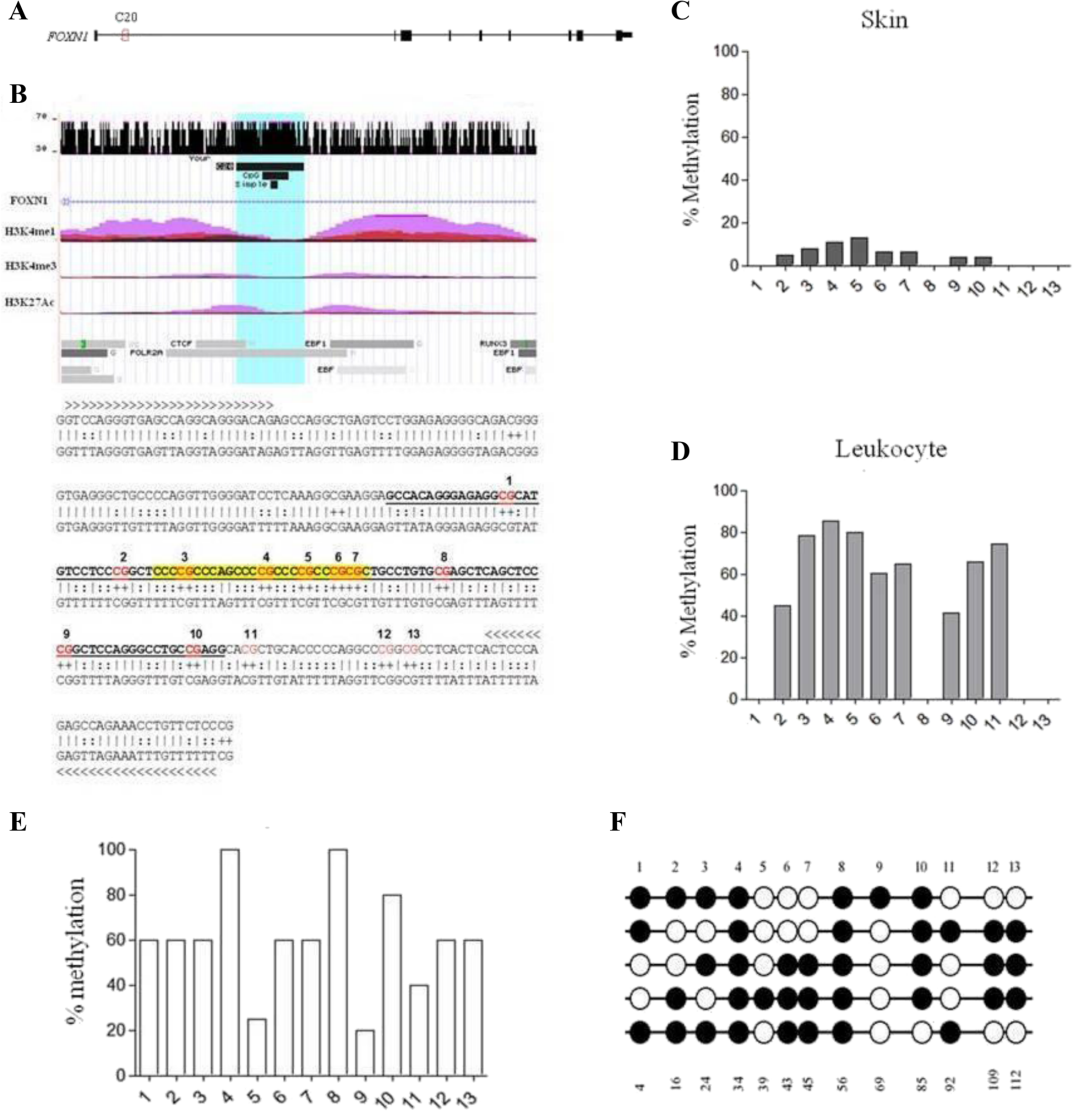
FOXN1 genomic context, potential methylation substrate C20 and CpG methylation status on hTEC. Panel (**a**) shows the genomic localization of C20 region on *FOXN1* gene and (**b**) (upper panel) presents the relative GC content, CpG island prediction, repetitive region (simple), histone modifications and transcriptional factor binding sites on C20 region. Histone modifications and transcriptional factor binding sites were evaluated by ENCODE consortium laboratories using chromatin immunoprecipitation followed by DNA sequencing [39]. The CpG island was predicted using MethPrimer web-based software [68] and the repetitive region was obtained in the *RepeatMasker* database [74]. Lower panel in (**b**) shows the C20 nucleotide sequence. Above is the normal genome sequence and below is the bisulfite converted sequence. In red is presented the CpG residues. The arrows (> and <) indicate the forward and reverse specific bisulfate primers used for sequencing. The CpG island is underlined in black and the yellow label marks the repetitive region. CpG methylation status on C20 region was obtained for FOXN1expressing human skin (**c**) and FOXN1 non-expressing human leukocytes (**d**) from the ENCODE database on DNA Methylation by Reduced Representation Bisulfite Sequencing data provided by ENCODE/HudsonAlpha laboratory. FOXN1 gene graphic representation and DNA elements regulation were adapted from UCSC genome browser from the human genome assembly hg19 using “ENCODE regulation” track features [39, 67]. The graph in (**e**) represents the percentage of CpG methylation, for each residue, and in hTEC cell line clone sequences, while the diagrams in (**f**) show the 13 CpG-s presented as lollipop diagrams, where the methylated residue is shown in black circles, while the non-methylated residue is in open circles. Percent values were obtained after multiple sequence alignment using ClustalW algorithm followed for methylated CpG quantification using *BiQ analyzer* software

As the hTEC line was refractive to *FOXN1* transcriptional induction, we thought to investigate DNA methylation, a possible epigenetic silencing mechanism, in hTEC line. Based on reported DNA methylation data from Encyclopedia of DNA elements (ENCODE) consortium, we first identified several CpG methylation sites, potentially involved in transcriptional regulation of the *FOXN1* gene. In the data set, there are 139 candidate residues for CpG methylation within the *FOXN1* gene. The candidate CpG residues are distributed in the promoter region, in exons 1, 2, 3, 7, 8, 9 and in introns 1, 2, 3, 6, 7 e 8. We chose one candidate region to test differential methylation: (i) because of overlap with *FOXN1* regulatory regions, (ii) the high GC % context, (iii) the presence of a predicted CpG island (Fig. 5a and b). The selected region is a 259 bp-sequence located at the first *FOXN1* intron, was termed C20 (Fig. 5a). The C20 region is close to the predicted *FOXN1* promoter and it satisfies all criteria for being “CpG methylation target” for gene expression regulation, such as high GC content and being within a CpG island. Specifically, the C20 sequence contains a simple repeat (CCCG)_n=3_ of 27 bp inside a CpG island of 100 bp-s (Fig. 5b, upper panel). Experimental data from ENCODE demonstrated that C20 is located within regulatory regions defined by histone modifications and it is included in a DNA-binding site cluster for RNA polymerase and the zinc-finger protein CTCF (*CCCTC-binding factor*) (Fig. 5b). There are 13 potential CpG methylation sites in this genomic sequence (Fig. 5b, low panel). Remarkably, as deduced from the ENCODE database [39], 8 of the 13 CpG cytosine residues show differential methylation pattern with respect to FOXN1 expressing human skin [25, 40] and FOXN1 non-expressing human leukocytes [41] as shown on Fig. 5c and d, respectively.

### FOXN1/C20 region CpG methylation status of hTEC line

We tested CpG methylation status of the 13 cytosine nucleotides present in the C20 sequence by bisulfite conversion, followed by PCR fragment cloning and DNA sequencing (See Additional file 1: Figure S4). Methylation status deduced from sequence analysis of 10 independent clones of the C20 region of hTEC cell line resembles of the FOXN1 non-expressing skin, suggesting that at least in the case of hTEC (Fig. 5e and f), CpG methylation within the C20 region correlates with the observed lack of expression and resistance to induction.

## Discussion

Immunosenescence is a physiological process characterized by the decline of both adaptive and innate immune functions [42]. The decreased ability to respond to antigens, at least in human, is thought to be the result of decreasing naïve T cell export that ultimately limits peripheral T cell repertoire in adults [43]. However, maintenance of naïve T cell repertoire in the periphery is thought to involve homeostatic regulation of proliferation [14, 44, 45]. Naïve T-cells derive from the thymus, however, histological signs of thymic involution are evident already at the first year of life [8]. As the organ ages, the thymic interstitium and ultimately intrathymic TEC areas are infiltrated by adipose tissue and peripheral lymphoid cells resulting in decreased thymopoiesis, eventually limiting the peripheral naïve T cell repertoire [16].

Little is known about the molecular mechanisms underlying thymic involution. Current reviews in the literature disagree whether thymic involution is organ autonomous, or is it due exclusively to decreasing function of TECs, or, and in addition, whether it involves changes in bone marrow derived stem cell activity and migration [14, 46]?

To address molecular mechanisms involved in human thymic involution during aging, first we tested gene expression levels of candidate genes in thymic biopsy specimen from days-old 5 to 78 years-old (*n* = 15) from consented cardiac surgery patients and in a human TEC line (hTEC). We choose *DLL1, DLL4, FOXN1* and *WNT4* genes for the analysis as these genes have been reported to be necessary for inducing intrathymic T cell development in mouse models [17, 36, 47, 48]. Particularly, *FOXN1* was selected because of reports indicating reduced expression in the ageing mouse thymus [26] and because recently, Bredenkamp and co-workers (2014) [18] showed that mouse embryonic fibroblasts transfected with inducible mouse *Foxn1* support T cell development *in vitro* and *in vivo.* Moreover, critical pathways for thymic development involve NOTCH ligands and receptors. In mouse models it was demonstrated that DLL1, DLL4, *Jagged-*1 (JAG-1) e JAG-2 ligands are expressed on TEC with their receptors expressed on thymocytes [49–51]. It is well established that DLL4 produced by TEC is responsible for T cell lineage commitment, being necessary for the maintenance of the three dimensional architecture of the thymic microenvironment [37, 47, 52]. Despite the broad range of studies on NOTCH and its ligands, little is known about their participation in thymic involution, especially in the human thymus. In addition to the NOTCH family of ligands, we also evaluated *WNT-4* gene expression. WNT-4 glycoprotein is secreted and it is produced both by TEC and thymocytes [53]. In a mouse model, WNT-4 was shown to play important role in organ development, T lymphocyte differentiation, and it was also associated with thymic involution [27, 48, 54, 55]. The WNT-4 glycoprotein also operates on TEC through induction of *Foxn1* expression in the mouse [19]. The decrease in the expression of FOXN1 has been studied as a hallmark of thymic senescence, although nothing is known on its expression in the human thymus during aging.

Our quantitative PCR gene expression experiments indicate that *DLL1* and *DLL4* are constitutively expressed in the human thymus, with a significant increase in the “Adult” group of thymus donors. In contrast, studies in experimental mouse models showed decrease in expression of *Dll4* in cortical TEC from old animals [31]. Since TEC isolation in our samples was not possible, it is reasonable to think that the high expression of *DLL1* and *DLL4* observed in “Adult” group is related to the presence of adipocytes in these samples. In fact, DLL4 expression has been reported in adipose tissue from human and animals subjected to high-fat diet [56]. Nevertheless, due to their increasing expression, DLL1 and DLL4 are not likely to limit human thymopoesis. Similarly to the NOTCH ligands, *WNT-4* gene is transcriptionally active in the human thymus in all tested groups, with a slight increase in expression in the “Child-adolescent” thymus samples. Although gene expression studies from whole organ are not suitable to address cell type specific expression, we suggest that the soluble factor, WNT-4, may be present in the aging thymus, thus it is not likely to limit thymopoetic activity either.

For the first time, we report here a striking three-fold decrease of *FOXN1* expression over time in the human thymus, when comparing the “Postnatal” group with the “Adult” group. In fact, the decrease of TEC associated expression levels may be markedly higher, as due to the relatively lower lymphocyte content of the aging thymus, the relative abundance of TEC is increasing. We thus suggest that *FOXN1* expression may limit thymopoiesis and its reduced expression may be responsible for thymic senescence. This notion is strongly supported by the fact that, although TECs are present in the samples of typical morphologically-defined aging thymus, FOXN1 protein were not detected in epithelial cells. The notion is also in-line with recent reports indicating that inducible *Foxn1* expression in mouse embryonic fibroblasts mediates trans-differentiation to functional TEC [18], suggesting that, at least in mice, FOXN1 is necessary and seemingly sufficient to induce thymopoiesis. Key role of *FOXN1* gene in human thymopoiesis is likely as the phenotype of the *FOXN1* null mutant humans [25, 57] is very similar to that of the nude phenotype of homozygous *Foxn1* mutant mice and rats [22, 24]. Moreover, thymic histology of *Foxn1* homozygous mutants strongly resembles that of thymic samples from aging individuals [28, 29].

To model age-related changes, we tested a human thymus derived epithelial cell line, hTEC, for expression of *FOXN1*, *DLL1* and *DLL4* genes. Although, the hTEC does bind to early thymocyte subsets, it does not induce or support full scale T cell development [33]. As we found no detectable *FOXN1* expression in cultured hTEC (while *DLL1* and *DLL4* gene expression were definitely detected) we tested whether hTEC cells would respond to stimuli reported to induce FOXN1 expression. To this end, we used 3D culture conditions shown to induce FOXN1 expression in hTEC cultures [37] and LiCl treatment, which was also reported to induce downstream canonic pathway of WNT signaling, including *Foxn1* expression induction in murine TEC [19]. The hTEC cell line did not respond to the tested specific stimulatory signals by increasing FOXN1 expression. We therefore suggest that although WNT-4 may regulate FOXN1 expression, it requires *FOXN1* gene to be responsive for inductive signaling by WNT-4. Thus hTEC is seemingly resistant to *FOXN1*-inducing stimuli. Among others, such resistance could be the result of transcriptional silencing, mediated by epigenetic regulatory mechanisms.

One of the most important epigenetic mechanisms that is often involved in transcriptional regulation during development is CpG methylation [58]. To investigate the methylation status of CpG residues in the FOXN1 gene in the hTEC, we tested a candidate regulatory region (we named C20) by bisulfite conversion followed by DNA sequencing. In the C20 candidate region FOXN1 expressing skin cells [25, 39, 40] show minimal methylation in 8 of the 13 candidate CpGs of the C20 region, while FOXN1 non-expressing leukocyte [39, 41] is highly methylated. We could observe that hTEC line DNA exhibits remarkably elevated methylation compared to skin DNA (from the ENCODE database). Overall methylation is decreasing with age, and we have detected this in the human thymic biopsy samples (ongoing studies; see Additional file 1: Figure S5) consistently with the age-dependent demethylation of CpG-s that has been reported in many tissues [59–61]. Hypermethylation of the C20 segment of the hTEC provides a strong clue supporting our hypothesis, namely that hypermethylation may gradually silence the *FOXN1* ultimately leading to decreased thymopoesis.

## Conclusions

For the first time in human thymus we show that FOXN1 expression decreases with age. Considering the central role of FOXN1 in thymopoesis we suggest that mechanism affecting FOXN1 expression regulation may be critically involved in thymic senescence. To support the notion we show that *FOXN1* gene in hTEC line is resistant to transcriptional induction that mimic physiological conditions. With respect to the hTEC line, our data supports the hypothesis that resistance of the *FOXN1* gene to physiological and chemical stimulatory signals may be mediated by an epigenetic mechanism, namely CpG methylation of specific regulatory regions(s) like C20. Although, the depth of the experiments and the heterogeneous tissue are severely limiting our conclusions, as a working hypothesis for future experimentation, we suggest that developmentally regulated CpG methylation of *FOXN1* may be a critical molecular mechanism behind thymic senescence and age related immunodeficiency. As our hypothesis needs further support from more accessible and specific rodent and in-vitro models, we plan to continue towards further understanding of epigenetic mechanisms behind thymic senescence with the hope that we may open avenues for therapeutic modulation via small molecule drugs.

## Methods

### Cell culture

The human TEC line (hTEC) was originally obtained from an infant thymus by primary explant culture and limiting dilution cloning [33], however since its derivation the cell lines has been passaged over 100 times. It has been kindly provided by Dr. Maria Luiza Toribio (*Universidad Autonoma de Madrid*, Madrid, Spain). These cells were shown to express cytokeratins and several surface proteins, such as CD71, CD40, MHC I (HLA-ABC), MHC II (HLA-DR)^low^, ICAM-1, LFA-3, CD44, integrin-type ECM receptors including VLA-4, VLA-5 and VLA-6 [32, 62]. Cells were cultured in 10 % fetal calf serum (Cultilab, São Paulo, Brazil) supplemented RPMI 1640 medium (Mediatech, Virginia, USA) at 37 °C in a 5 % CO_2_ atmosphere. Alternatively, these cells were cultured in three-dimensional cell culture system using macroporous cellulose microcarriers (Cytopore™, Asaki Kasei Medical Co., GE Healthcare, Japan) as described previously [63] with some modifications. Briefly, 100 μL of phosphate-buffered saline (PBS)-hydrated macroporous cellulose microcarriers were colonized with cells in a volume of the 2x10^5^ cells under nitrocellulose membrane from Transwell™ inserts (Costar, Corning Incorporated, USA) and cultured for 4 days with complete medium at 37 °C in a 5 % CO_2_ atmosphere. Under these conditions, the cells were allowed to grow in 3D environment (Fig. 3a).

### Sample collection and histology

Thymus samples were obtained from consented paediatric and adult patients undergoing cardiac surgery at the György Gottsegen National Institute for Cardiology, (Semmelweis University, Budapest, Hungary) and from the Department of Cardiac Surgery of the Clinical Center, University of Debrecen (Debrecen, Hungary) under a protocol approved by the Hungarian Ethics Committee for Science and Research (11739-/2014/EKU 107/2014.) and the local institutional review boards. Patients’ blood counts were in the normal range in all cases as this is pre-requisite condition for selected eletive surgeries. Fresh thymus fragments were cleaned, washed in PBS and fixed in neutral buffered formalin for routine histological examination. Additional 3×3×3 mm fragments were snap frozen and kept at −80 °C until use.

### Lithium chloride treatment

hTEC line were cultured under standard conditions with complete medium for two days, and then treated with 20 mM of lithium chloride (LiCl) for 6 and 18 h as previously described to induce the WNT signaling pathway [19, 64]. Non-treated cells and sodium chloride (NaCl)-treated cells were used as negative controls. After treatment, cells were used for RNA isolation or fixed with 100 % methanol for imunofluorescence staining.

### Immunofluorescence

Immunofluorescence assays were used to evaluate FOXN1, cytokeratin and β-catenin protein expression in thymus samples and hTEC line cultured under various conditions. Briefly, thymic frozen sections fixed in acetone, and hTEC cultures fixed with 100 % methanol, were re-hydrated in PBS and incubated for 30 min with PBS containing 1 % bovine serum albumin to block unspecific binding. Next, the specimens were incubated with primary antibodies specific for human FOXN1 (donkey IgG, 1:50; Santa Cruz Biotechnology, Heidelberg, Germany), pan-cytokeratin (rabbit IgG, 1:100; DAKO, Aligent Technologies, Glostrup, Denmark) or human β-catenin (mouse monoclonal antibody, IgG_1_, 1:30; Santa Cruz Biotechnology, Heidelberg, Germany) for 1 h and 30 min at room temperature in a humidified chamber. After washings with PBS, the slides were incubated with corresponding fluorochrome-labeled secondary antibody for 45 min at room temperature, in a dark humidified chamber. Secondary reagents were: goat anti-rabbit (1:400; Molecular Probes, Life Technologies), goat anti-mouse (1:400; Molecular Probes, Life Technologies) and donkey anti-goat (1:400; Invitrogen). Next, after washing in PBS, the specimens were mounted for examination in the fluorescence microscope, a Zeiss Axio Imager A2 (Carl Zeiss, Oberkochen, Germany) equipped with Axio Vision Release 4.8.2 software (Zeiss). Negative controls in which primary antibodies were replaced by unrelated immunoglobulins or in which the secondary antibody was used alone did not generate significant immunolabeling.

### RNA isolation, cDNA synthesis and PCR assays

Total RNA from thymus samples and hTEC line were purified by phenol-chloroform and used as templates for first strand cDNA synthesis followed by gene expression analysis via RT-PCR and quantitative PCR assays as follows. Small fragments of frozen thymi were homogenised with tissue homogenizer (T10 Basic, IKA®) in 1 ml of Trizol (TriReagent®, Molecular Research Center, Ohio, USA) whereas hTEC cultured in conventional monolayers or in 3D scaffolds were released from the substrate with trypsin/EDTA treatment for 10 min, centrifuged, washed in PBS and homogenized with 1 ml of Trizol (TriReagent®, Molecular Research Center, Ohio, USA). After homogenization, RNA was purified according manufacturer’s instructions. Total RNA concentration and purity were determined by the ratio of absorbance readings at 260 vs. 280 nm on NanoDrop ND 2000 spectrometer (Thermo Scientific, Delaware, USA). Up to 2 micrograms of total RNA were used to synthesize cDNA using high capacity cDNA reverse transcription kit (Applied Biosystems, California, USA) following manufacturer’s protocol. Quantitative PCR and RT-PCR reactions were performed using oligonucleotides previously described in the literature or specifically designed based on the target gene sequences reported in NCBI GenBank using the Primer3 software from the NCBI/BLAST platform [65]. Specific sequences are shown in Table 1. For RT-PCR reactions, eight microliters of diluted cDNA (1:5) were mixed with nuclease-free water, 1.5 millimolar of MgCl_2,_ 1X Taq polimerase buffer, 0.025 millimolar of dNTPs, 0.05 units per microliter of Taq polimerase and 0.5 micromolar of each primer in a 50 μl of total volume (all from Applied Biosystems, California, USA except the nuclease-free water and primers). The cycling conditions were as follows: initial denaturation at 94 °C for 2 min, followed by 30 cycles with denaturation 94 °C for 30 s, annealing at 60 °C for 30 s, and elongation at 72 °C for 30 s using the A&B thermocycler 2720 (Applied Biosystems, California, USA). Ten microliters from each PCR reaction were run on 2 % agarose gels at 90 V. The PCR products were visualized under UV light using ethidium bromide staining. The quantitative PCR reactions were performed using 5 μl from 5-fold diluted cDNA sample mixed with Maxima SYBR Green/ROX qPCR Master Mix (Thermo Scientific, California USA) containing specific oligonucleotides (0.3 micromolar; Table 1) in microwells of optical 384-well plates according to manufacturer’s protocol. All samples were run in triplicates. The reactions were run in the ABI PRISM® 7900HT sequence analyser instrument (Applied Biosystems, California, USA) with Sequence Detector System 2.2 Software following three-step cycling protocol with 95 °C for 10 min for initial denaturation; 95 °C for 15 s to denaturation; 60 ° C of 30 s to elongation and 30 s of 72 °C to extension. After amplification, the Ct values were used to obtain normalized expression values as previously described [66].

**Table 1:** Forward and reverse primer sequences used on the quantitative PCR and RT-PCR assays

Target	Sequence forward (5'-3')	Sequence reverse (5'-3')	Amplicon size (pb)	Reference
*FOXN1* (NM_003593.2)	TCCCTCACTCACTGACTTCG (1628–1647)	GTGGCATCGAAGATGATGTC (1746–1727)	119	[72]
*DLL1* (NM_005618.3)	TGCAACCAGGACCTGAACTA (1323–1342)	CTCCGTTCTTACAAGGGCTG (1491–1472)	163	*
*DLL4* (NM_019074.3)	CAGAGTGTCGGATATCAGCG (2288–2307)	CTCCTGCCTTATACCTCCGT (2402–2383)	115	*
*WNT-4* (NM_030761.4)	CAGCAGAGCCCTCATGAACC (647–666)	GCCAGCACGTCTTTACCTCACA (768–747)	122	[73]
*HPRT-1* (NM_000194.2)	CCTGGCGTCGTGATTAGTG (183–201)	TCGAGCAAGACGTTCAGTCC (320–301)	138	*
*TFRC* (NM_001128148.1)	CTAGTGTTCTTCTGTGTGGCAGTT (115–138)	ACAATGGTTCTCCACCAAACAAG (197–175)	83	*
*RPL13A* (NM_001270491.1)	CGGACCGTGCGAGGTATGCT (244–263)	AGCAGGAACCACCATCCGCT (366–347)	123	*

HPRT-1 (hypoxanthine phosphoribosyltransferase 1), TRFC (transferrin receptor) and RPL13A (ribosomal protein L13a) were used as control housekeeping genes. (*)Designed using the software Primer3 [37]. FOXN1: forkhead box N1; DLL1: Delta-like 1 (Drosophila); DLL4: Delta-like (Drosophila) 4; WNT-4: wingless-type MMTV integration site family, member 4.

### DNA Isolation and bisulfite treatment

Genomic DNA samples were isolated from the hTEC line using the Wizard® Genomic DNA Purification Kit (Promega, Wisconsin, USA) according to the manufacturer’s instructions. After isolation, DNA concentration and purity were determined by the ratio of absorbance readings at 260 and 280 nm on NanoDrop ND 2000 spectrometer (Thermo Scientific, Delaware, USA). Two micrograms of DNA were used to perform bisulfite treatment using Epitect Bisulfite Kit (Qiagen, Hilden, Germany) following the supplier’s instructions. After reaction, the converted DNA was purified using silica columns (EpiTect spin columns, Qiagen, Hilden, Germany), eluted in RNAse and DNAse free water and keep in −20 freezer until use.

### DNA Methylation analysis

Candidate regulatory sequences for CpG methylation were selected from the *FOXN1* gene using features of the University of California Santa Cruz (UCSC) genome browser (UCSC ID: uc010crm.3; [67]). For selection, in addition to known features relevant to probable CpG methylation, we used experimental data reported in UCSC genome browser, namely the DNA methylation, by reduced representation bisulfite sequencing performed by the Encyclopedia of DNA Elements (ENCODE) consortium [39]. Selected regions were exploited as targets to design bisulfite-treated DNA specific oligonucleotides using the MethylPrimer Express v 1.0 software (Applied Biosystems) and the web-based software MethPrimer [68]. Both programs have algorithms for primer design specific for bisulfite-treated DNA, according the criteria described by Li and Dahiya (2002) [68]. For bisulfite sequencing, isolated DNA from hTEC line were converted with sodium bisulfite using Epitect Bisulfite kit (Qiagen, Hilden, Germany) as described above. The selected candidate sequence (Fig. 5a and b) was amplified with specific primers for FOXN1/C20 region (F: GTTTAGGGTGAGTTAGGTAGGGATAG; R: AAAAACAAATTTCTAACTCTAAAAATAAAT). The PCR products were cloned in pGEM-T plasmid (pGEM®-T Easy Vector System I; Promega, Wisconsin, USA) and the inserts were sequenced using BigDye® Terminator Cycle Sequencing Kit (Applied Biosystems, California, USA) using forward or reverse PCR primers. The reactions were performed in ABI3730xl DNA analyzer (Applied Biosystems, California, USA) at the Fiocruz DNA sequencing platform (Rio de Janeiro, Brazil). Traces were analysed with BioEdit: Sequence Alignment Editor v. 7.2.5 (Ibis Biosciences, Califorina, USA), aligned with ClustalW v1.83 [69] and submitted to CpG quantification using BiQ analyzer v2.00 [70]. Percent methylation, with respect to each individual CpG, and the global average percentage methylation per each candidate sequences were displayed. For methylation call we used >90 % conversion threshold. Lollipop diagrams were made per samples using Quantification Tool for Methylation Analysis (QUMA) web software [71].

### Statistical analyses

To test the probability of significant differences among the different study groups, the data obtained were analyzed GraphPad Prism software version 5.00 (GraphPad Prism Software, Inc.) using the non-parametric one-way ANOVA Kruskal-Wallis test, followed by Dunn’s post-test. The values were represented by the mean ± standard error of the mean (SEM) and considered significant when p ≤ 0.05.

## Additional file

Additional file 1 **Supplementary material.** (PDF 867 kb)

## Abbreviations

TCR: T cell repector
TEC: Thymic epithelial cell
ECM: Extracellular matrix
MHC: Major histocompatibility complex
FOXN1: forkhead box N1
DLL1: Delta-like 1 (Drosophila)
DLL4: Delta-like (Drosophila) 4
WNT-4: wingless-type MMTV integration site family, member 4
JAG: Jagged
SV40: Simius virus 40
PAX-1: Paired box gene 1
LiCl: Lithium chloride
NaCl: Sodium chloride
ENCODE: Encyclopedia of DNA Elements
UCSC: Unversity of California Santa Cruz
