# Supplementary material for: Decline of FOXN1 gene expression in human thymus correlates with age: possible epigenetic regulation
Source: Immun Ageing. 2015 Oct 29;12:18. doi: 10.1186/s12979-015-0045-9 (PMC4625732; doi:10.1186/s12979-015-0045-9)
Supplement: Supplementary file 1 — Supplementary material. (PDF 867 kb) [file 12979_2015_45_MOESM1_ESM.pdf]

Supplementary material

Supplementary table 1 – Normalized expresssion values per sample, mean and standard deviation of Forkhead box N1 (*FOXN1*) gene.

FOXN1				
**Group**	**Sample Age**	**Normalized gene expression value**	**Mean**	**SD per group**
**Posnatal**	5 days-old	0,266258729	0,2940	0,06941
	1 year-old	0,242802884		
	1 year-old	0,373019084		
**Child-Adolescent**	10 years-old	0,240222927	0,2534	0,02782
	17 years-old	0,285327986		
	14 years-old	0,234575866		
**Adult**	49 years-old	0,067409705	0,1056	0,04663
	57 years-old	0,113462441		
	59 years-old	0,122242775		
	66 years-old	0,054146431		
	75 years-old	0,170809434		

Supplementary table 2 – Non-parametric One-Way ANOVA (Kruskal-Wallis test) followed by Dunn's multiple comparison test of normalized expresssion values of *FOXN1* gene.

Non-parametric One-Way ANOVA - *FOXN1* expression			
**Kruskal-Wallis test**			
P value	0,0195		
Exact or approximate P value?	Gaussian Approximation		
P value summary	*		
Do the medians vary signif. (P < 0.05)	Yes		
Number of groups	3		
Kruskal-Wallis statistic	7,879		
**Dunn's Multiple Comparison Test**	**Difference in rank sum**	**Significant? P < 0.05?**	**Summary**
Posnatal vs Child-Adolescent	1,667	No	Ns
Posnatal vs Adult	6,333	Yes	0,0195*
Child-Adolescent vs Adult	4,667	No	Ns

Supplementary table 3 – Non-parametric *t* test of normalized expresssion values of *FOXN1* gene.

Non-parametric 't" test - *FOXN1* expression	
**Mann Whitney test**	***p* value (two-tailed)**
Posnatal vs Child-Adolescent	0,4000
Posnatal vs Adult	0,0357*
Child-Adolescent vs Adult	0,0357*

Supplementary figure 1 - Spearman linear regression analysis of *FOXN1* expression data set. A: regression plot, B: statistical summary, C: input data. The results show that the data is statistically significant in fact without age grouping.

A

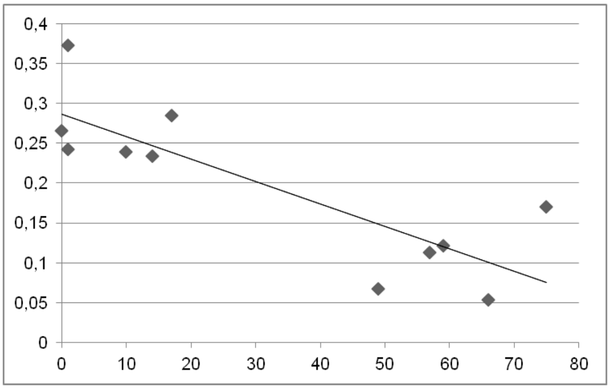

B

Age	FOXN1 normalized expression values
0,01	0,266258729
1	0,242802884
1	0,373019084
10	0,240222927
17	0,285327986
14	0,234575866
49	0,067409705
57	0,113462441
59	0,122242775
66	0,054146431
75	0,170809434

C

Number of XY Pairs	11
Spearman r	-0,7517
95% confidence interval	-0.9342 to -0.2573
P value (two-tailed)	0,0098
P value summary	**
Exact or approximate P value?	Exact
Is the correlation significant? (alpha=0.05)	Yes

Supplementary table 4 – Normalized expresssion values per sample, mean and standard deviation (SD) of Delta-like 1 (*DLL1*) gene.

DLL1				
**Group**	**Sample age**	**Normalized expression value**	**Mean**	**SD per group**
**Posnatal**	5 days-old	0,070445716	0,05495	0,03622
	1 year-old	0,098867962		
	1 year-old	0,02614221		
	1 year-old	0,024350335		
**Child-Adolescent**	7 years-old	0,08201404	0,1605	0,09197
	10 years-old	0,084885469		
	17 years-old	0,266056597		
	14 years-old	0,209041312		
**Adult**	49 years-old	0,17860024	0,5932	0,4084
	57 years-old	1,199760048		
	66 years-old	0,269715036		
	75 years-old	0,742344941		
	78 years-old	0,575636735		

Supplementary table 5 – Non-parametric One-Way ANOVA (Kruskal-Wallis test) followed by Dunn's multiple comparison test of normalized expresssion values of *DLL1* gene.

**Non-parametric One-Way ANOVA - *DLL1* expression**			
Kruskal-Wallis test			
P value	0,0139		
Exact or approximate P value?	Gaussian Approximation		
P value summary	*		
Do the medians vary signif. (P < 0.05)	Yes		
Number of groups	3		
Kruskal-Wallis statistic	8,558		
**Dunn's Multiple Comparison Test**	**Difference in rank sum**	**Significant? P < 0.05?**	**Summary**
Posnatal vs Child-Adolescent	-3,500	No	ns
Posnatal vs Adult	-7,600	Yes	0,0139*
Child-Adolescent vs Adult	-4,100	No	ns

Supplementary table 6 – Non-parametric *t* test of normalized expresssion values of *DLL1* gene.

**Non-parametric 't" test - *DLL1* expression**	
**Mann Whitney test**	***p* value (two-tailed)**
Posnatal vs Child-Adolescent	0,1143
Posnatal vs Adult	0,0159*
Child-Adolescent vs Adult	0,0635

Supplementary table 7 – Normalized expresssion values per sample, mean and standard deviation (SD) of Delta-like 4 (*DLL4*) gene.

DLL4				
**Group**	**Sample age**	**Normalized expression value**	**Mean group**	**SD per**
**Posnatal**	1 year-old	0,060775	0,06134	0,006366
	1 year-old	0,055274		
	1 year-old	0,067968		
**Child-Adolescent**	7 years-old	0,384012	0,4568	0,07953
	10 years-old	0,43852		
	17 years-old	0,570134		
	14 years-old	0,434496		
**Adult**	49 years-old	0,480831	0,7606	0,3624
	57 years-old	0,293505		
	57 years-old	0,922305		
	66 years-old	0,622254		
	75 years-old	0,959233		
	78 years-old	1,285413		

Supplementary table 8 – Non-parametric One-Way ANOVA (Kruskal-Wallis test) followed by Dunn's multiple comparison test of normalized expresssion values of *DLL4* gene.

**Non-parametric One-Way ANOVA - *DLL4* expression**			
Kruskal-Wallis test			
P value	0,0205		
Exact or approximate P value?	Gaussian Approximation		
P value summary	*		
Do the medians vary signif. (P < 0.05)	Yes		
Number of groups	3		
Kruskal-Wallis statistic	7,775		
**Dunn's Multiple Comparison Test**	**Difference in rank sum**	**Significant? P < 0.05?**	**Summary**
Posnatal vs Child-Adolescent	-4,750	No	ns
Posnatal vs Adult	-7,667	Yes	0,0205
Child-Adolescent vs Adult	-2,917	No	ns

Supplementary table 9 – Non-parametric *t* test of normalized expresssion values of *DLL4* gene.

**Non-parametric 't" test - *DLL4* expression**	
**Mann Whitney test**	***p* value (two-tailed)**
Posnatal vs Child-Adolescent	0,0571
Posnatal vs Adult	0,0238*
Child-Adolescent vs Adult	0,1714

Supplementary table 10 – Normalized expresssion values per sample, mean and standard deviation (SD) of Wingless-type MMTV integration site family, member 4 (*WNT4*) gene.

WNT-4				
**Group**	**Sample Age**	**Normalized expression value**	**Mean**	**SD per group**
**Posnatal**	5 days-old	0,446514	0,3564	0,1295
	1 year-old	0,207967		
	1 year-old	0,414811		
**Child-Adolescent**	7 years-old	0,431773	0,5333	0,1531
	10 years-old	0,409908		
	17 years-old	0,744537		
	14 years-old	0,546798		
**Adult**	49 years-old	0,145398	0,3843	0,2591
	57 years-old	0,293505		
	57 years-old	0,173372		
	75 years-old	0,73409		
	78 years-old	0,575031		

Supplementary table 11 – Non-parametric One-Way ANOVA (Kruskal-Wallis test) followed by Dunn's multiple comparison test of normalized expresssion values of *WNT4* gene.

Non-parametric One-Way ANOVA - WNT-4 expression			
Kruskal-Wallis test			
P value	0,4931		
Exact or approximate P value?	Gaussian Approximation		
P value summary	ns		
Do the medians vary signif. (P < 0.05)	No		
Number of groups	3		
Kruskal-Wallis statistic	1,414		
**Dunn's Multiple Comparison Test**	**Difference in rank sum**	**Significant? P < 0.05?**	**Summary**
Posnatal vs Child-Adolescent	-2,583	No	ns
Posnatal vs Adult	0,06667	No	ns
Child-Adolescent vs Adult	2,650	No	ns

Supplementary table 12 – Non-parametric *t* test of normalized expresssion values of *WNT4* gene.

Non-parametric 't" test - WNT-4 expression	
**Mann Whitney test**	***p* value (two-tailed)**
Posnatal vs Child-Adolescent	0,4000
Posnatal vs Adult	1,0000
Child-Adolescent vs Adult	0,4127

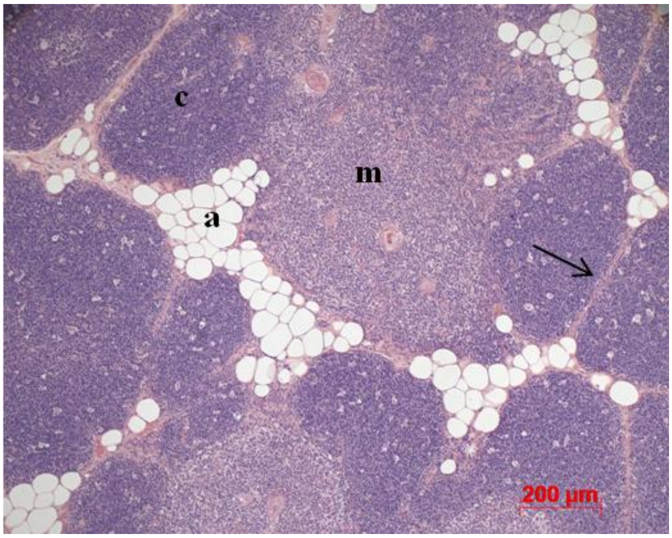

Supplementary figure 2 – Human thymus histology. Photomicrograph show morphological aspects of a thymus sample from 14 years-old donor ("Child-Adolescent" group). Paraffin sections were stained with haematoxilin-eosin stain and analyzed by light microscopic examination. (4x, objective magnification). c= cortical region; m= medullary region; a= Adipose tissue; arrow= connective tissue septum.

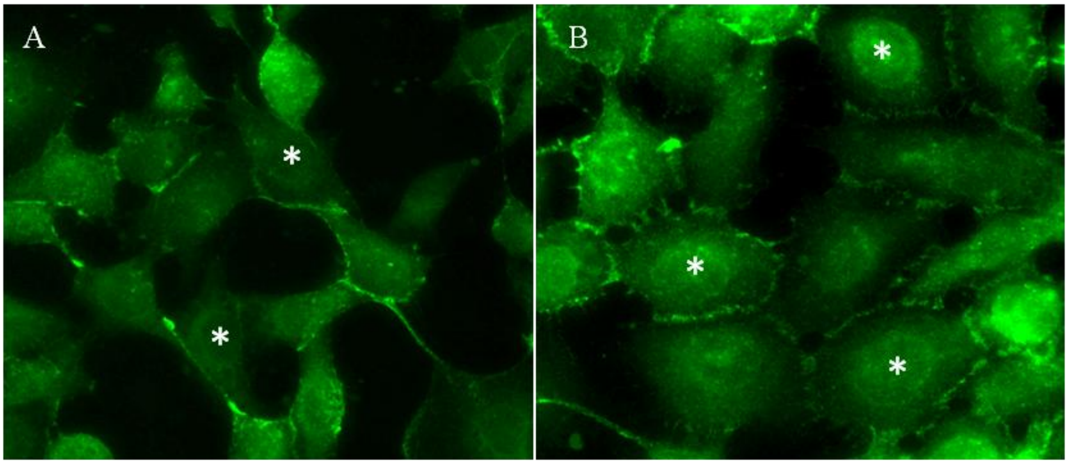

Supplementary figure 3 – Beta-catenin nuclear translocation in hTEC after treatment with LiCl for 6 hrs. Non-treated TEC monolayer (**A**) and LiCl-treated cells (**B**) were incubated with a monoclonal anti-β-catenin antibody followed by secondary reaction with Alexa 488 conjugated anti-mouse Ig antibody. (*) β-catenin staining in hTEC nucleus. 40x, objective magnification.

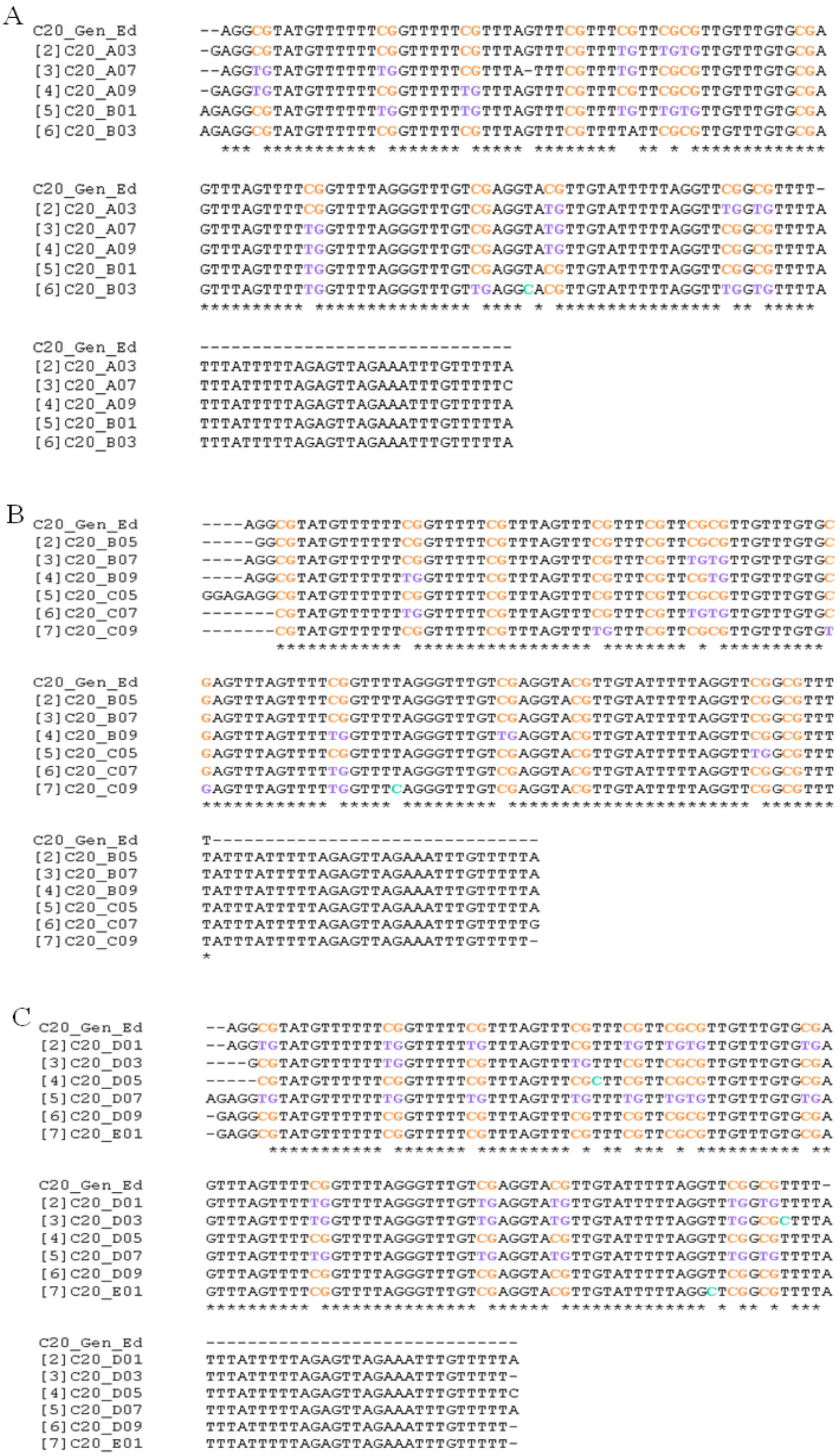

Supplementary figure 4– Sequence alignment from FOXN1/C20 gene region. Panels show the multiple alignment of the sequence clones on FOXN1/C20 region from bisulfite-converted DNA fragments. DNA samples were obtained from hTEC (**A**), and thymus samples from 5 days-old (**B**) and 59 years-old donors (**C**).

(legend continues at the next page).

Methylated CpGs are represented in orange (CG), while in purple are presented the non-methylated CpG (TG), characterized by citosin conversion in timin during bisulfite treatment followed by PCR. Non-converted citosins are showed in green. The multiple alignment was performed with ClustalW 1.83, inclued in the BiQ analyzer package.

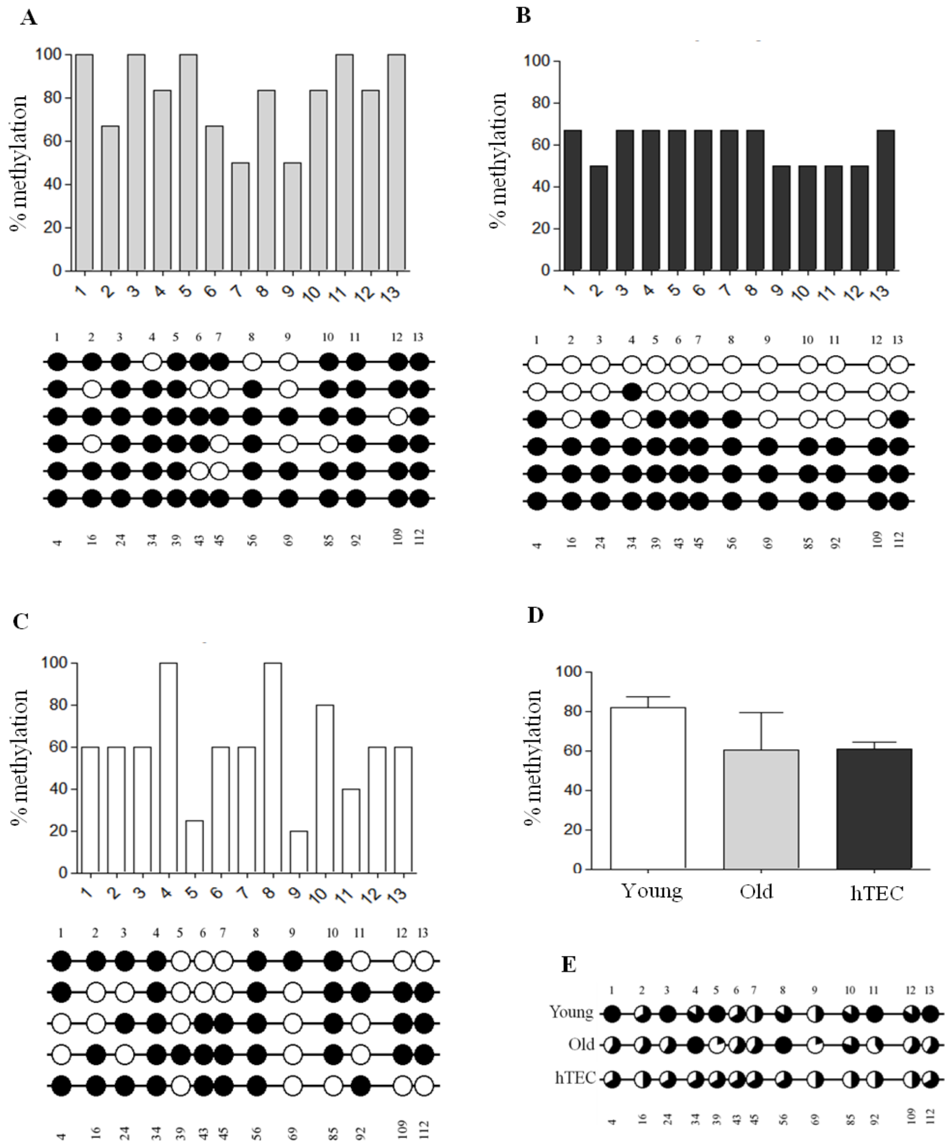

Supplementary figure 5 – FOXN1/C20 region CpG methylation status on human thymic samples. Graphs represents the percentage of CpG methylation, for each residue, in young thymus (5 days-old; “Postnatal” group) (**A-**)**,** old thymus (59 years-old ; “Adult” group) (**B**), and in hTEC cell line (**C**) clone sequences. Lower panels (**A-C**) show the 13 CpG-s presented as lollipop diagrams, where the methylated residue is shown in black circles, while the non-methylated residue is in open circles.

(legend continues at the next page).

Panel **D** represents the percent mean SEM of CpG methylation per clone sequence for each sample and **E** shows the percent mean per each residue per sample expressed as small pie-charts in the lollipop diagrams. The black label represents the percent mean of methylated CpG and white label is marked for percent mean of unmethylated CpG. Percent values were obtained after multiple sequence alignment using ClustalW algorithm followed for methylated CpG quantification using *BiQ analyzer* software. Difference between the samples was analyzed through Kruskal-Wallis non-parametric test followed by Dunn’s multiple test.
